# Molecular-based race classification of *Pyrenophora tritici-repentis* causing tan spot of wheat in Japan

**DOI:** 10.1270/jsbbs.23012

**Published:** 2023-10-28

**Authors:** Keita Kato, Yusuke Ban, Mikiko Yanaka, Shoya Kitabayashi, Hiroyuki Sekiguchi, Keisuke Tomioka, Miwako Ito

**Affiliations:** 1 Western Region Agricultural Research Center (Kinki, Chugoku and Shikoku Regions), National Agriculture and Food Research Organization (NARO), Fukuyama, Hiroshima 721-8514, Japan; 2 Kyushu-Okinawa Agricultural Research Center, NARO, Chikugo, Fukuoka 833-0041, Japan; 3 Institute for Plant Protection, NARO, Tsukuba, Ibaraki 305-8666, Japan

**Keywords:** *Pyrenophora tritici-repentis*, tan spot, race classification, *Tox* gene, bread wheat, durum wheat

## Abstract

Tan spot, a foliar disease of *Triticum* spp. such as bread wheat (*T. aestivum* L.) and durum wheat (*T. turgidum* ssp. *durum* (Desf.) Husn.) caused by the filamentous fungus *Pyrenophora tritici-repentis* (Died.) Drechsler leads to serious losses of crop yield and quality in some areas in Japan. *P. tritici-repentis* is classified into eight races according to the combinations of three necrotrophic effectors, PtrToxA, PtrToxB, and PtrToxC encoded by *ToxA*, *ToxB*, and *ToxC1*, respectively. Race classification has been based on reaction of a differential variety to necrotrophic effectors, which is tested by inoculation. Recent identification of the *Tox* genes and development of specific DNA markers have enabled us to classify races of *P. tritici-repentis* collected in Japan by *Tox* gene genotyping. We found that 17 strains collected from *Triticum* spp. in Japan were mainly race 1 or 2, because they carried *ToxA* as a toxin gene by current race classification; wheat genotype *tsn1* is resistant to *ToxA*. Establishment of wheat cultivars carrying *tsn1* would be most effective for decreasing agronomic losses caused by the disease in Japan.

## Introduction

A foliar disease of wheat (*Triticum* spp.) caused by the filamentous fungus *Pyrenophora tritici-repentis* (Died.) Drechsler, called tan spot or yellow spot. It occurs in almost all wheat-growing regions all over the world including Japan (Kamel *et al.* 2019, Lamari and Strelkov 2010, Nishi *et al.* 1993, Shi *et al.* 2022, Tsukiboshi 2005, Yoshimatsu and Kato 2003). The disease can lead to considerable yield loss up to 50% and/or red smudge on grains to deteriorate their quality on susceptible cultivars to the disease in favorable condition (Lamari and Bernier 1989, Rees *et al.* 1982, Schilder and Bergstrom 1994). Recently, the wheat cultivated area of Japan is about 220,000 ha, and yield is about 1 million t a year (https://www.maff.go.jp/j/tokei/kouhyou/sakumotu/index.html, accessed April 7, 2023). Tan spot has become a serious problem in some areas of Japan. For reducing damages by the disease, crop rotation with rice, harvested residue removal and fungicides application are mainly done, but the most effective method for reducing disease is the development of genetically resistant varieties (Kariyawasam *et al.* 2018, Running *et al.* 2022).

The pathogenic fungus is currently known to have eight races defined according to combinations of three necrotrophic effectors, PtrToxA (13.2 kDa), PtrToxB (6.6 kDa), and PtrToxC, encoded respectively by *ToxA*, *ToxB*, and *ToxC* (Ballance *et al.* 1996, Ciuffetti *et al.* 1997, Effertz *et al.* 2002, Faris *et al.* 2013, Martinez *et al.* 2004, Strelkov *et al.* 2006). PtrToxA induces necrosis, and PtrToxB and PtrToxC induce chlorosis. Races of *P. tritici-repentis* have been identified on the basis of necrotrophic effector production and virulence after inoculation of a set of four differential varieties (Abdullah *et al.* 2017a, 2017b, 2017c, Faris *et al.* 2013, Kamel *et al.* 2019, Lamari and Strelkov 2010). Recently, genes encoding *ToxA*, *ToxB*, *toxb*, and *ToxC1* have been identified. A *ToxB* homolog, *toxb* has missense mutations and encodes a non-toxic protein (Kamel *et al.* 2019, Strelkov *et al.* 2006). PtrToxC is considered to be a low-molecular-mass protein (Effertz *et al.* 2002), and a conserved hypothetical gene, *ToxC1*, is required but is not sufficient for PtrToxC production (Shi *et al.* 2022). Specific DNA markers for these genes have been developed (Kamel *et al.* 2019, Shi *et al.* 2022).

Although information about *Tox* genes is available, knowledge of *P. tritici-repentis* races in Japan is insufficient. In this study, we attempted to classify the strains present in Japan into races by genetic methods.

## Materials and Methods

### Fungus materials and genomic DNA extraction

We used 17 strains isolated from bread wheat (*T. aestivum* L.), durum wheat (*T. turgidum* ssp. *durum* (Desf.) Husn.), or *Agropyron* sp. registered as *P. tritici-repentis* at the Research Center of Genetic Resources, National Agriculture and Food Research Organization (NARO) (Table 1). Each strain was grown in potato dextrose broth (Becton, Dickinson and Co., Franklin Lake, NJ, USA), and genomic DNA was extracted in DNAs-ici-F extraction buffer (Rizo Inc., Tsukuba, Japan).

### PCR amplification and sequencing

The species identity of the 17 strains was verified according to Marin-Felix *et al.* (2019) using DNA sequences of the internal transcribed spacer region (ITS) and the coding regions of the genes for glyceraldehyde-3-phosphate dehydrogenase (*gapdh*) and the second largest subunit of RNA polymerase II (*rpb2*). These regions were amplified in a PCR Thermal Cycler Dice (Takara Bio, Shiga, Japan) using AmpliTaq Gold 360 (Applied Biosystems, Waltham, MA, USA) at an initial 95°C for 10 min; 35 cycles of 95°C for 30 s, 58°C for 30 s, and 72°C for 60 s; and a final 72°C for 7 min. PCR primers are listed in Table 2. The PCR fragments were separated by electrophoresis in 2% agarose gel, visualized with Gel Red stain (Biotium, Fremont, CA, USA), and extracted with a Qiaquick Gel Extraction Kit (Qiagen, Hilden, Germany). They were sequenced by Fasmac Co. Ltd. (Kanagawa, Japan). The sequences were registered in the DNA Data Bank of Japan (DDBJ) with the accession numbers shown in Table 1.

### Phylogenetic analysis

A phylogenetic tree was constructed using the UPGMA algorithm with 1000 bootstrap replicates in MEGA11 software (Tamura *et al.* 2021) with the sequences of the 17 strains and *P. tritici-repentis* CBS 191.29, *P. grahamii* CBS 128044, *P. dictyoides* CBS 127933, *P. cynosuri* CBS 127918, and *P. fugax* CBS 509.77 (Marin-Felix *et al.* 2019). A pairwise distances were calculated among 17 strains in MEGA11 software.

### Genotyping of *Tox* genes

Genomic DNA was amplified in a Thermal Cycler Dice using Quicktaq HS (Toyobo, Osaka, Japan) by quadruplex PCR (*ToxA*, *ToxB*, *toxb*, and *chitin synthase 1* (*CHS1*) as an internal control) or duplex PCR (*ToxB*, *toxb*, or *ToxC1* and *CHS1* at an initial 95°C for 2 min, followed by 30 cycles of 94°C for 30 s, and 68°C for 60 s. The amplified PCR fragments were separated and visualized as above.

## Results

### Species identification

**Fig. 1.**
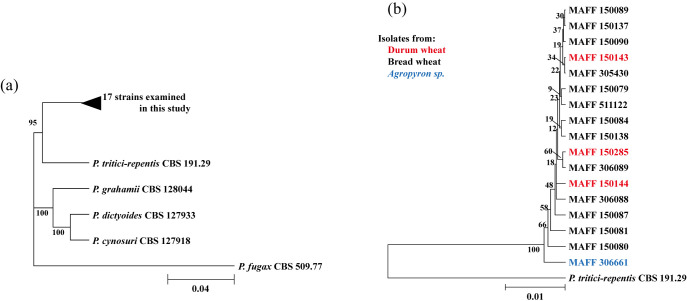
Phylogenetic analysis of the 17 strains examined in this study. The DNA sequences of ITS, *gapdh*, and *rpb2* (Table 1) of each isolate were aligned, and phylogenetic trees were generated in MEGA11 software with the UPGMA algorithm and 1000 bootstrap replicates. GenBank accession numbers: *P. tritici-repentis* CBS 191.29 (ITS, MK540018; *gapdh*, MK540229; *rpb2*, MK540144); *P. grahamii* CBS 128044 (ITS, MK539988; *gapdh*, MK540197; *rpb2*, MK540113); *P. dictyoides* CBS 127933 (ITS, MH877971; *gapdh*, MK540191; *rpb2*, MK540109); *P. cynosuri* CBS 127918 (ITS, MK539980; *gapdh*, MK539980; *rpb2*, MK540106); *P. fugax* CBS 509.77 (ITS, MK539985; *gapdh*, MK540194; *rpb2*, MK540111). (a) Overall view of the phylogenetic tree. (b) Enlarged view of the subclade containing the 17 strains and *P. tritici-repentis* CBS 191.29. Bootstrap values are indicated at each branch node, and the scale bar indicates the number of nucleotide substitutions per site.

All 17 strains listed in Table 1 formed a subclade together with *P. tritici-repentis* CBS 191.29 and were thereby confirmed as *P. tritici-repentis* (Fig. 1a). The pairwise distance was ranged 0.0005–0.0094 (data not shown). The minimum was between MAFF 150089 and MAFF 150137, and the maximum was between MAFF 150080 and MAFF 306661. The positions of the 17 strains within the subclade did not reflect the wheat species or cultivar from which they were isolated, or the collection site, and year (Fig. 1b, Table 1).

### *Tox* gene distribution

**Fig. 2.**
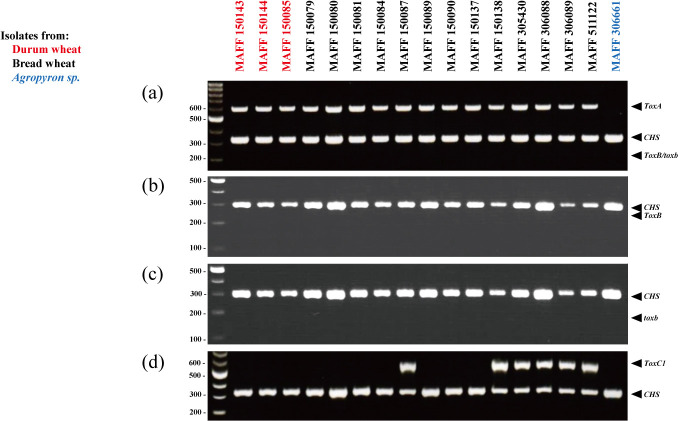
PCR-based genotyping of *Tox* genes. The MAFF numbers of the strains are shown on the top. A 275-bp fragment of the *CHS1* gene was amplified as an internal positive control. (a) Quadruplex PCR of *ToxA* (573 bp) and *ToxB/toxb* (232 bp). (b) Duplex PCR of *ToxB* (245 bp). (c) Duplex PCR of *toxb* (157 bp). (e) Duplex PCR of *ToxC1* (505 bp).

The 16 strains collected from bread wheat or durum wheat had *ToxA*-specific fragments, but not *ToxB*- and *toxb*-specific fragments in quadruplex or duplex PCR (Fig. 2a–2c, Table 1). The 6 strains of them, MAFF 150087, 150138, 305430, 306088, 306089 and 511122, also had *ToxC1*-specific fragments. *ToxA* is necessary and sufficient for production of PtrToxA, and *ToxC1* is necessary but not sufficient for that of PtrToxC. So, PtrToxC is unproduced through expression of *ToxC1* only. According to the current race classification by Faris *et al.* (2013), we inferred that the 10 strains of MAFF 150143, 150144, 150085, 150079, 150080, 150081, 150084, 150089, 150090 and 150137 with *ToxA*-specific fragments only belong to race 2 producing PtrToxA only, as well as that the 6 strains with *ToxA*- and *ToxC1*-specific fragments belong to race 2 or race 1 producing both PtrToxA and PtrToxC. MAFF 306661 collected from *Agropyron* sp. did not have *ToxA*-, *ToxB*-, *toxb*-, and *ToxC1*-specific fragments. We inferred that it belongs to race 4, which has no PtrToxs (Table 1).

## Discussion

This is the first report of identification of tan spot pathogens in Japan as *P. tritici-repentis* by current genetic methods and the inference of their races from the presence of *Tox* genes (Figs. 1, 2, Table 1). 17 strains were identified as *P. tritici-repentis* with ITS, *gapdh* and *rpb2* sequence by current phylogenetic analysis according to Marin-Felix *et al.* (2019), a pairwise distances of them were very low and not characterized them by wheat species or cultivar from which they were isolated, or the collection site and year (Fig. 1, Table 1). No diversity was found ITS and *β-tubulin* gene in 10 strains of *P. tritici-repentis* from bread wheat in Mie Prefecture in Japan, although isolated year, site, and cultivar were differed (Hafez *et al.* 2022). We deduced from these reports that identification of *P. tritici-repentis* could be succeeded, but not clustered the collected information of 17 strains by phylogenetical analysis.

A race distribution of *P. tritici-repentis* in the world without East Asia including Japan was reported that race 1 was predominant in the Americas, Europe, North and South Asia and the Caucasus (Kamel *et al.* 2019). And race 2 was second predominant in the Americas and North and South Asia, but it was low frequency in Europe and the Caucasus (Kamel *et al.* 2019). In Australia and New Zealand, only races 1 or 2 were distributed (Antoni *et al.* 2010, Kamel *et al.* 2019). In Africa, a tendency of a race distribution of *P. tritici-repentis* were different from other areas, race 5 was predominant and race 6 was second predominant (Kamel *et al.* 2019). Hafez *et al.* (2022) used inoculation to analyze 10 isolates of *P. tritici-repentis* from bread wheat grown in Mie Prefecture in Japan and classified 8 isolates as race 1 and 2 isolates as race 2. Based on molecular analysis, we presume that the predominant race of *P. tritici-repentis* isolated from not only bread wheat but also durum wheat in Japan is race 1 or race 2, each of which has at least the *ToxA* gene and distributed in west area from Kanto region (Table 1, Fig. 2). Especially, 10 strains classified as race 2 were distributed around Hiroshima and Okayama Prefectures (Table 1, Fig. 2), because there is possibility that they were collected from a relatively confined location. In addition, this study inferred that MAFF 306661 from *Agropyron* sp. in Hokkaido was race 4. Race 4 has already been found from noncereal grasses in various areas in America with low frequency (Ali and Francl 2003). In the case of a distribution of wheat yellow mosaic virus in Japan, geographically distribution was found that pathotype I, II, and III were distributed in central, northern, and southern areas of Japan, respectively (Ohki *et al.* 2014). So, due to verify race identification with more strains of *P. tritici-repentis* from various collected sites and host wheats may lead complete understanding of race distribution in Japan.

The pathosystem between *P. tritici-repentis* and wheat has been studied in detail. In particular, hypersensitive reaction of the host leading to necrosis is mediated by PtrToxA and depends on the presence or absence of *Tsn1* (an S/TPK-NBS-LRR gene) in the host (Faris *et al.* 2010). Wheat genotypes with *Tsn1* are sensitive to PtrToxA, whereas null (*tsn1*) genotypes are insensitive (Faris *et al.* 2010). PtrToxC interacts with the wheat sensitivity gene *Tsc1* to induce chlorosis, and the *tsc1* genotype is insensitive (Effertz *et al.* 2002, Kariyawasam *et al.* 2018, Shi *et al.* 2022). Under the current distribution of *P. tritici-repentis* races, establishment of wheat cultivars with the *tsn1* genotype is strongly required and of those with the *tsc1* genotype is desirable as a direction of the crop breeding program to decrease agronomic losses caused by tan spot in Japan. In addition to introduce *tsn1* and *tsc1* in Japanese wheat cultivar(s) under the current situation, pyramiding of PtrToxB insensitive wheat genotype *tsc2* with them might be better to be prepared for future invasions of new races of *P. tritici-repentis*. This breeding strategy will be possibility to confer a combined insensitivity for both necrosis and chlorosis by any PtrToxs.

## Author Contribution Statement

All authors contributed to the study conception and design. KK, KT and HS prepared the materials. KK, YB, MY and KT collected and analyzed the data. All authors interpreted the data. KK and KT wrote the initial draft and all authors reviewed and approved the manuscript.

## Figures and Tables

**Table 1.** Materials used in this study

MAFF number	Collection		Host	Cultivar of isolation	Accession number*^a^*				PCR amplification of *Tox* genes*^b^*				Predicted race
	Location (town/city, prefecture)	Year			ITS	*gapdh*	*rpb2*		*ToxA*	*ToxB*	*toxb*	*ToxC1*	
150143	Fukuyama, Hiroshima	2017	Durum wheat	Setodure	LC685424	LC685441	LC685458		+	–	–	–	Race 2
150144	Fukuyama, Hiroshima	2018	Durum wheat	Setodure	LC685425	LC685442	LC685459		+	–	–	–	Race 2
150085	Tsuyama, Okayama	2017	Durum wheat	Setodure	LC685426	LC685443	LC685460		+	–	–	–	Race 2
150079	Miyoshi, Hiroshima	2016	Bread wheat	Minaminokaori	LC685427	LC685444	LC685461		+	–	–	–	Race 2
150080	Higashi-Hiroshima, Hiroshima	2016	Bread wheat	Kinuhime	LC685428	LC685445	LC685462		+	–	–	–	Race 2
150081	Tsuyama, Okayama	2016	Bread wheat	Setokirara	LC685429	LC685446	LC685463		+	–	–	–	Race 2
150084	Tsuyama, Okayama	2018	Bread wheat	Setokirara	LC685430	LC685447	LC685464		+	–	–	–	Race 2
150087	Tsuyama, Okayama	2017	Bread wheat	Setokirara	LC685431	LC685448	LC685465		+	–	–	+	Race 1 or race 2
150089	Tsuyama, Okayama	2017	Bread wheat	Setokirara	LC685432	LC685449	LC685466		+	–	–	–	Race 2
150090	Tsuyama, Okayama	2018	Bread wheat	Setokirara	LC685433	LC685450	LC685467		+	–	–	–	Race 2
150137	Fukuyama, Hiroshima	2018	Bread wheat	Setokirara	LC685434	LC685451	LC685468		+	–	–	–	Race 2
150138	Fukuyama, Hiroshima	2018	Bread wheat	Chinese Spring	LC685435	LC685452	LC685469		+	–	–	+	Race 1 or race 2
305430	Karatsu, Saga	1977	Bread wheat	Unknown	LC685436	LC685453	LC685470		+	–	–	+	Race 1 or race 2
306088	Ibaraki Prefecture	1990	Bread wheat	Unknown	LC685437	LC685454	LC685471		+	–	–	+	Race 1 or race 2
306089	Mie Prefecture	1990	Bread wheat	Unknown	LC685438	LC685455	LC685472		+	–	–	+	Race 1 or race 2
511122	Karatsu, Saga	1977	Bread wheat	Unknown	LC685439	LC685456	LC685473		+	–	–	+	Race 1 or race 2
306661	Ashoro, Hokkaido	2002	*Agropyron* sp.	Unknown	LC685440	LC685457	LC685474		–	–	–	–	Race 4

^*a*^ DNA sequences were registered in DDBJ. ^*b*^ + *Tox* gene-specific primers amplified a fragment; – they did not amplify a fragment.

**Table 2.** Sequences of primers used in this study

Purpose and target gene	Sequence 5ʹ–3ʹ		Reference
For fungal identification			
Internal transcribed spacer (ITS)	TCCGTAGGTGAACCTGCGG	TCCTCCGCTTATTGATATGC	White *et al.* (1990)
*Glyceraldehyde-3-phosphatedehydrogenase* (*gapdh*)	CAACGGCTTCGGTCGCATTG	GCCAAGCAGTTGGTTGTGC	Berbee *et al.* (1999)
*Second largest subunit of RNA polymerase II* (*rpb2*)	GGTCGTGACGGTAAACTGG	ATCATGGCCGGATGAATCT	Designed in this study
For race identification by quadruplex PCR			
*ToxA*	GCGTTCTATCCTCGTACTTC	GCATTCTCCAATTTTCACG	Kamel *et al.* (2019)
*ToxB*	GCTACTTGCTGTGGCTATC	ACTAACAACGTCCTCCACTTTG	Kamel *et al.* (2019)
*toxb*	GCTACTTGCTGTGGCTATC	TATGAATGATTGACTGGGGTTA	Kamel *et al.* (2019)
*CHS*	TGGGGCAAGGATGCTTGGAAGAAG	TGGAAGAACCATCTGTGAGAGTTG	Kamel *et al.* (2019)
For race identification by duplex PCR			
*ToxB*	GACTACCATGCTACTTGCTGTG	AACAACGTCCTCCACTTTGC	Kamel *et al.* (2019)
*toxb*	AAGTGGTCATTGCGACTGG	CCTCCACTTGCCAAACTCTC	Kamel *et al.* (2019)
*ToxC1*	GAGCAGCATTTTGACGAGTG	TGGAAGTCGTTCATTGTTGC	Shi *et al.* (2022)
